# Supplementary material for: Photonic-electronic integrated circuit-based coherent LiDAR engine
Source: Nat Commun. 2024 Apr 11;15:3134. doi: 10.1038/s41467-024-47478-z (PMC11009237; doi:10.1038/s41467-024-47478-z)
Supplement: Supplementary file 1 — Supplementary Information [file 41467_2024_47478_MOESM1_ESM.pdf]

Supplementary Information

Photonic-electronic integrated circuit-based coherent LiDAR engine

Anton Lukashchuk[1,*], Halil Kerim Yildirim[2,*], Andrea Bancora[1], Grigory Lihachev[1], Yang Liu[1], Zheru Qiu[1], Xinru Ji[1], Andrey Voloshin[1], Sunil A. Bhave[3], Edoardo Charbon[2,†], Tobias J. Kippenberg[1,†]

[1]*Institute of Physics, Swiss Federal Institute of Technology Lausanne (EPFL), CH-1015 Lausanne, Switzerland*

[2]*Advanced Quantum Architecture Laboratory (AQUA), Switzerland, Swiss Federal Institute of Technology Lausanne (EPFL), CH-2002 Neuchâtel, Switzerland*

[3]*OxideMEMS Lab, Purdue University, 47907 West Lafayette, IN, USA*

[*] *These authors contributed equally to this work*

[†] *Corresponding Authors: Tobias J. Kippenberg - tobias.kippenberg@epfl.ch, Edoardo Charbon - edoardo.charbon@epfl.ch*

	**This work** [Arbitrary*/ Sawtooth**]	[1]	[2]	[3]	[4]
Process	0.13 µm BiCMOS	0.18 µm CMOS	0.18 µm HV-BCD	0.35 µm HVCMOS	Discrete IGBTs
HV technology / supply	No	No	Yes	Yes	Yes
Arbitrary Waveform generation	Yes	No	No	Yes	Yes
Architecture	Charge Pump	Stacked switches	Current Driver	Current Driver	H bridge Multilevel
V_{pp} [stdev***]	22.3 V [93 mV] / 15.4 V [46.5mV]	13.2 V	60 V	19.75 V	28.8 kV
Frequency [stdev***]	13.7 kHz [36.6 Hz] / 43.1 kHz [81.2 Hz]	5 MHz	7 MHz	-	20 kHz
Load	Capacitive 26 pF	Capacitive 15.4 pF	Capacitive 15 pF	Resistive 10 kΩ	Series RC R_s= 6kΩ C_s= 32 pF
Power consumption	1.28 mW**** / 2.35 mW****	12.8 mW	6.26 mW	-	480 W

*Arbitrary waveform in Fig. 2c second from top. **Sawtooth waveform in Fig. 2d.
***Measured over 20k samples. ****Power consumption of AWG with 26 pF load.

Supplementary Figure 1: HV-AWG comparison table [1–4]

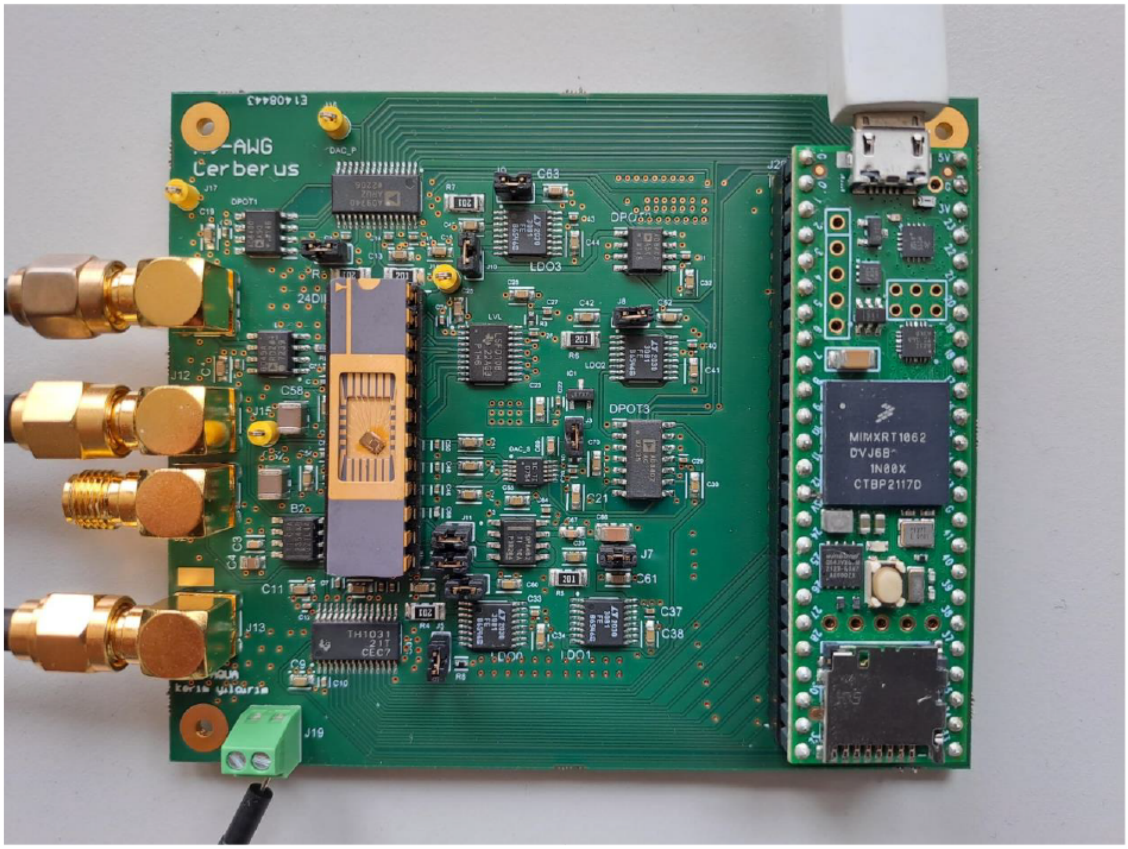

Supplementary Figure 2: Printed circuit board (PCB) of the HV-AWG The output voltage is connected to a unity-gain voltage buffer on the PCB through the package, and the buffer output is used to drive the SMA connector that is used for the measurements.

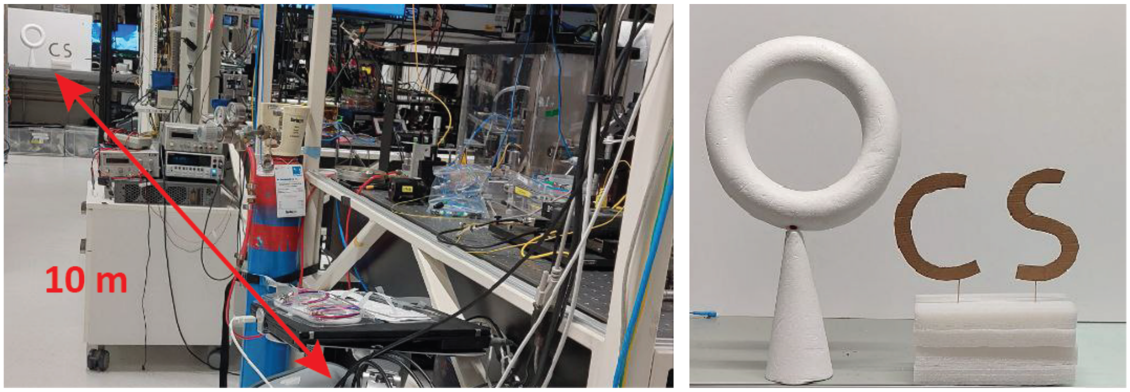

Supplementary Figure 3: Imaging scene.

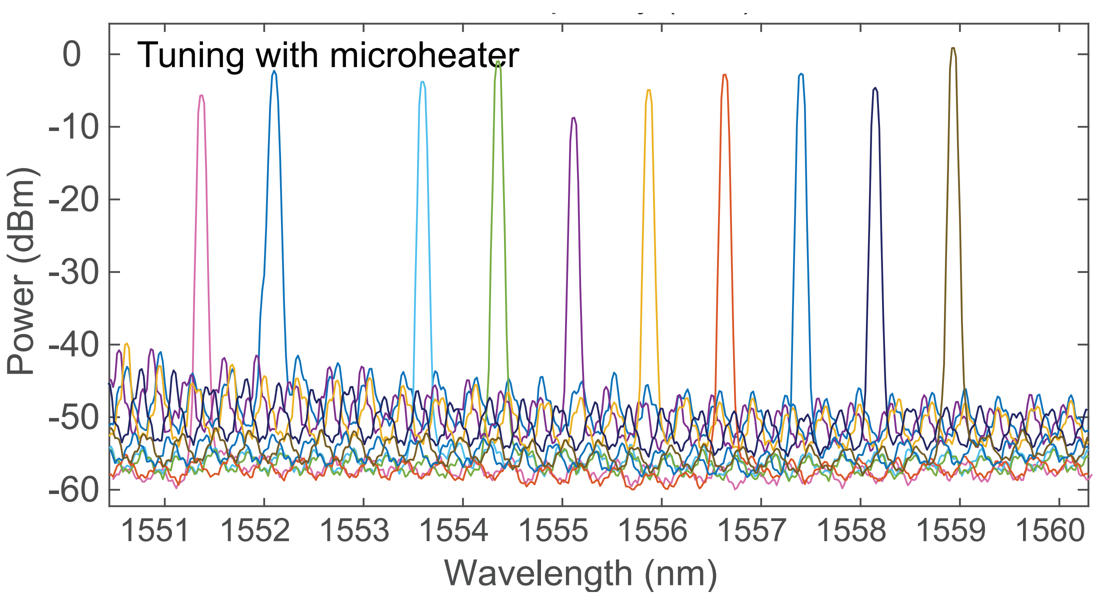

Supplementary Figure 4: Optical spectra of Vernier laser emission at different microheater power with 30 mW electrical power steps.

References

1. Lee, J. *et al.* A 36-channel auto-calibrated front-end asic for a pmut-based miniaturized 3-d ultrasound system. *IEEE Journal of Solid-State Circuits* **56**, 1910–1923 (2021).

2. Jung, G. *et al.* Single-chip reduced-wire active catheter system with programmable transmit beamforming and receive time-division multiplexing for intracardiac echocardiography. In *2018 IEEE International Solid-State Circuits Conference-(ISSCC)*, 188–190 (IEEE, 2018).

3. Sooksood, K., Noorsal, E., Becker, J. & Ortmanns, M. A neural stimulator front-end with arbitrary pulse shape, hv compliance and adaptive supply requiring 0.05 mm 2 in 0.35 μm hvcmos. In *2011 IEEE International Solid-State Circuits Conference*, 306–308 (IEEE, 2011).

4. Dragonas, F. A., Neretti, G., Sanjeevikumar, P. & Grandi, G. High-voltage high-frequency arbitrary waveform multilevel generator for dbd plasma actuators. *IEEE Transactions on Industry Applications* **51**, 3334–3342 (2015).
